# Complete Genome Sequencing of a Multidrug-Resistant and Human-Invasive *Salmonella enterica* Serovar Typhimurium Strain of the Emerging Sequence Type 213 Genotype

**DOI:** 10.1128/genomeA.00663-15

**Published:** 2015-06-18

**Authors:** Edmundo Calva, Claudia Silva, Mussaret B. Zaidi, Alejandro Sanchez-Flores, Karel Estrada, Genivaldo G. Z. Silva, Luz M. Soto-Jiménez, Magdalena Wiesner, Marcos Fernández-Mora, Robert A. Edwards, Pablo Vinuesa

**Affiliations:** a Departamento de Microbiología Molecular, Instituto de Biotecnología, Universidad Nacional Autónoma de México, Cuernavaca, Morelos, México; b Microbiology Research Laboratory, Hospital General O’Horan and Infectious Diseases Research Unit, Hospital Regional de Alta Especialidad de la Península de Yucatán, Mérida, Yucatán, México; c Unidad Universitaria de Apoyo Bioinformático, Universidad Nacional Autónoma de México, Cuernavaca, Morelos, México; d Computational Science Research Center, San Diego State University, San Diego, California, USA; e Department of Computer Science, San Diego State University, San Diego, California, USA; f Mathematics and Computer Science Division, Argonne National Laboratory, Argonne, Illinois, USA; g Centro de Ciencias Genómicas, Programa de Ingeniería Genómica, Universidad Nacional Autónoma de México, Cuernavaca, Morelos, México

## Abstract

*Salmonella enterica* subsp. *enterica* serovar Typhimurium strain YU39 was isolated in 2005 in the state of Yucatán, Mexico, from a human systemic infection. The YU39 strain is representative of the multidrug-resistant emergent sequence type 213 (ST213) genotype. The YU39 complete genome is composed of a chromosome and seven plasmids.

## GENOME ANNOUNCEMENT

An epidemiological surveillance program in Mexico showed that *Salmonella enterica* serovar Typhimurium was the most frequently isolated serovar from human infections (1). The multilocus sequence type 213 (ST213) was assigned to more than half of the Typhimurium population. This genotype was associated with food animal samples, lacked the *Salmonella* virulence plasmid, and carried multidrug resistance IncA/C plasmids (2, 3). Most of the systemic infections recorded during the surveillance period were caused by ST213 strains. Strain YU39 was isolated from the blood culture of an 8-year-old child displaying hepatomegaly and thrombocytopenia (3). This strain was studied for its capacity for conjugative transfer of the resistance *bla*_CMY-2_ gene through interactions between IncA/C and IncX1 plasmids (4).

Genomic DNA was extracted by standard protocols (5) and sheared into ~10-kb fragments for RSII-PacBio library preparation and P5-C3 sequencing. The continuous long read (CLR) data were assembled using the HGAP/Quiver protocol (SMRT portal version 2.2.0) (6). This resulted in an assembly containing eight contigs with ~70× genome coverage. A final polishing step was performed by remapping quality-filtered (7), 72-bp-long Illumina reads (*n* = 9,857,489, originating from a 200-bp paired-end library sequenced in a GAII instrument) and 454 GS FLX+ single-end reads (*n* = 58,117; mean length, 418.04) onto the assembly using BWA (8), increasing its coverage to >150×. The aligned reads were converted to BAM format with SAMtools (9), and passed down to Pilon (10) to correct for small indels and SNPs. SMRTview analysis of CLRs mapped at the contig ends in the SMRT portal revealed their circular structure. Terminal repeats were trimmed with Minimus2 (11). The total size of the resulting genome assembly is 5,190,370 bp, with a G+C content of 51.94%, consisting of a 4.89-Mb chromosome and seven plasmids (156.3, 88.9, 42.2, 5.1, 4.8, 4.2, and 2.7 kb). Gene calling and annotation was performed with a modified version of the Prokka annotation pipeline (12). A total of 5,216 genes were identified, including 89 tRNAs, 22 rRNAs, 1 transfer-messenger RNA (tmRNA), 174 noncoding RNAs (ncRNAs), and 4,930 coding sequences (CDSs). Three CRISPR-CAS repeats and 453 signal peptides were annotated. The annotation was manually curated and enriched with predictions from the PHAST server (13) to annotate prophages, and IslandViewer to annotate genomic islands (14).

Five complete prophages were located on the chromosome: ST104, Gifsy-2, P88-like, ST64B, and Gifsy-2, as well as several phage remnants. The three large plasmids of 156.3, 88.9, and 42.2 kb were assigned to the multidrug resistance pIncA/C, a phage-like plasmid, and the conjugative pIncX1, respectively. This is the first complete genome sequence of a Mexican pathogenic and multidrug-resistant *Salmonella* Typhimurium strain.

### Nucleotide sequence accession numbers. 

The complete genome sequences for the chromosome and the seven plasmids of *Salmonella* Typhimurium strain YU39 are available in GenBank under the accession numbers CP011428, CP011429, CP011430, CP011431, CP011432, CP011433, CP011434, and CP011435.

## References

[1] Zaidi MB, Calva JJ, Estrada-Garcia MT, Leon V, Vazquez G, Figueroa G, Lopez E, Contreras J, Abbott J, Zhao S, McDermott P, Tollefson L 2008 Integrated food chain surveillance system for *Salmonella* spp. in Mexico. Emerg Infect Dis 14:429–435. doi:10.3201/eid1403.071057. 18325258 PMC2570816

[2] Wiesner M, Calva E, Fernandez-Mora M, Cevallos MA, Campos F, Zaidi MB, Silva C 2011 *Salmonella* Typhimurium ST213 is associated with two types of IncA/C plasmids carrying multiple resistance determinants. BMC Microbiol 11:9. doi:10.1186/1471-2180-11-9. 21223599 PMC3025833

[3] Wiesner M, Zaidi MB, Calva E, Fernández-Mora M, Calva JJ, Silva C 2009 Association of virulence plasmid and antibiotic resistance determinants with chromosomal multilocus genotypes in Mexican *Salmonella enterica* serovar typhimurium strains. BMC Microbiol 9:131. doi:10.1186/1471-2180-9-131. 19573249 PMC2715408

[4] Wiesner M, Fernández-Mora M, Cevallos MA, Zavala-Alvarado C, Zaidi MB, Calva E, Silva C 2013 Conjugative transfer of an IncA/C plasmid-borne *bla*_CMY-2_ gene through genetic re-arrangements with an IncX1 plasmid. BMC Microbiol 13:264. doi:10.1186/1471-2180-13-264. 24262067 PMC4222815

[5] Sambrook J, Russell DW 2001 Molecular cloning: a laboratory manual, 3rd ed. Cold Spring Harbor Laboratory Press, Cold Spring Harbor, NY.

[6] Chin CS, Alexander DH, Marks P, Klammer AA, Drake J, Heiner C, Clum A, Copeland A, Huddleston J, Eichler EE, Turner SW, Korlach J 2013 Nonhybrid, finished microbial genome assemblies from long-read SMRT sequencing data. Nat Methods 10:563–569. doi:10.1038/nmeth.2474. 23644548

[7] Bolger AM, Lohse M, Usadel B 2014 Trimmomatic: a flexible trimmer for Illumina sequence data. Bioinformatics 30:2114–2120. doi:10.1093/bioinformatics/btu170. 24695404 PMC4103590

[8] Li H, Durbin R 2010 Fast and accurate long-read alignment with Burrows-Wheeler transform. Bioinformatics 26:589–595. doi:10.1093/bioinformatics/btp698. 20080505 PMC2828108

[9] Li H, Handsaker B, Wysoker A, Fennell T, Ruan J, Homer N, Marth G, Abecasis G, Durbin R 2009 The Sequence Alignment/Map format and SAMtools. Bioinformatics 25:2078–2079. doi:10.1093/bioinformatics/btp352. 19505943 PMC2723002

[10] Walker BJ, Abeel T, Shea T, Priest M, Abouelliel A, Sakthikumar S, Cuomo CA, Zeng Q, Wortman J, Young SK, Earl AM 2014 Pilon: an integrated tool for comprehensive microbial variant detection and genome assembly improvement. PLoS One 9:e112963. doi:10.1371/journal.pone.0112963. 25409509 PMC4237348

[11] Treangen TJ, Sommer DD, Angly FE, Koren S, Pop M 2011 Next generation sequence assembly with AMOS. Curr Protoc Bioinformatics 33:Unit 11.8. doi:10.1002/0471250953.bi1108s33. PMC3072823 21400694

[12] Seemann T 2014 Prokka: rapid prokaryotic genome annotation. Bioinformatics 30:2068–2069. doi:10.1093/bioinformatics/btu153. 24642063

[13] Zhou Y, Liang Y, Lynch KH, Dennis JJ, Wishart DS 2011 PHAST: a fast phage search tool. Nucleic Acids Res 39(Suppl 2):W347–W352. doi:10.1093/nar/gkr485. 21672955 PMC3125810

[14] Dhillon BK, Chiu TA, Laird MR, Langille MG, Brinkman FS 2013 IslandViewer update: improved genomic island discovery and visualization. Nucleic Acids Res 41(W1):W129–W132. doi:10.1093/nar/gkt394. 23677610 PMC3692065

